# Supplementary material for: Ethical, Legal, and Social Assessment of AI-Based Technologies for Prevention and Diagnosis of Rare Diseases in Health Technology Assessment Processes
Source: Healthcare (Basel). 2025 Apr 4;13(7):829. doi: 10.3390/healthcare13070829 (PMC11988925; doi:10.3390/healthcare13070829)
Supplement: Supplementary file 1 [file healthcare-13-00829-s001.zip › healthcare-3547368-supplementary.pdf]

Supplementary Materials

File S1.

Literature review

What specific issues, beyond those addressed in the EUnetHTA Core Model®, should be considered in the ethical, legal, and social evaluation of technologies designed for rare diseases?

Research question

The research question was reformulated using the PICO model, which includes the study population (P), the intervention being evaluated (I), the comparator (C), and the outcome of interest (O). Table S1 describes the PICO model underlying this research.

Table S1 – PICO model

**Population**	Persons affected by rare diseases
**Intervention(s)**	Technologies dedicated to rare diseases
**Comparator(s)**	Not relevant
**Outcome (s)**	Ethical issues
	Legal issues
	Social issues

Search

We searched PubMed/MEDLINE for articles indexed, using the search string defined based on the outlined PICO model. We developed a search string comprising three semantic clusters: the first cluster addresses the topics of methodology, the second focuses on Health Technology Assessment (HTA), and the third pertains to ethical, legal, and social issues. The complete search string is presented in Table S2.

Table S2 – Search string

(((Framework* OR method* OR methodology OR guideline*) AND ("Technology Assessment, Biomedical"[Mesh])) AND ("Rare Diseases"[Mesh])) AND (ethic* OR social OR legal OR juridical)

Inclusion and exclusion criteria

The documents identified through the search strategy were considered eligible unless they met one or more of the following exclusion criteria:

- duplicate studies;
- studies involving a technology not under investigation;
- studies concerning health conditions not under investigation;

- studies involving a population not under investigation;
- study types not relevant for the analysis;
- insufficient information reported in the study on any of the investigated aspects;
- abstract/full-text not available;
- study not available in English.

The reference list of the included documents was manually checked for additional relevant studies. We did not assess the methodological quality of the included studies.

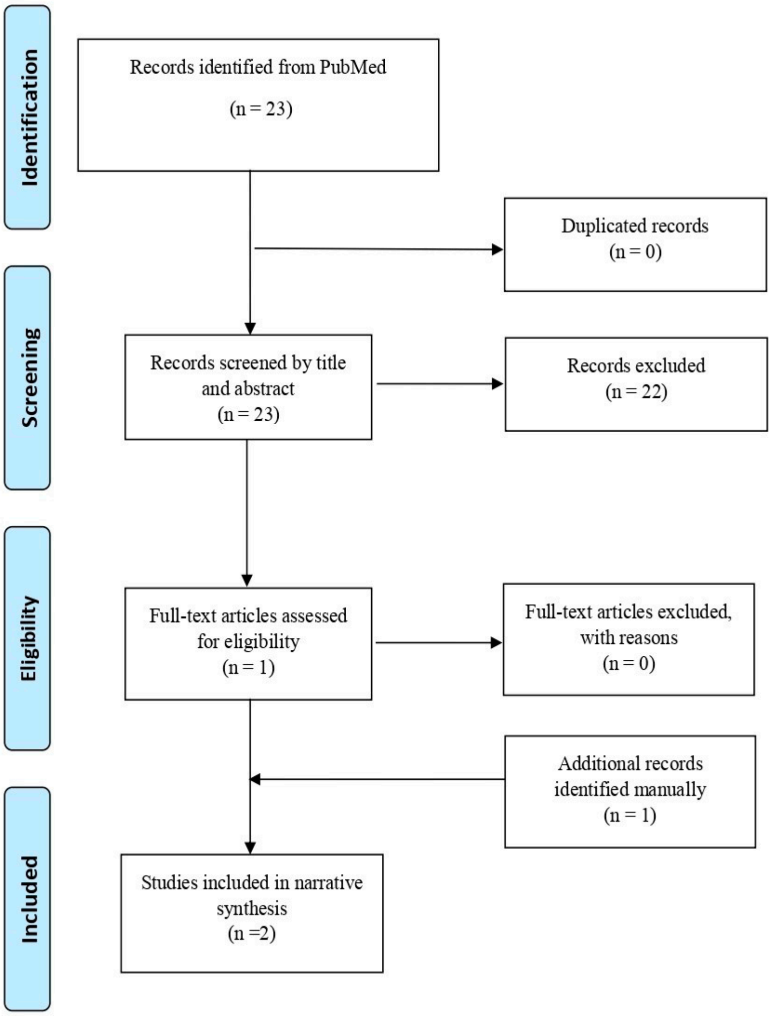

Figure S1 – Study selection

Table S3 – Data extraction

Source	Focus	General considerations	Ethical issues	Legal issues	Social issues
Nestler-Parr S, Korchagina D, Toumi M, Pashos CL, Blanchette C, Molsen E, Morel T, Simoens S, Kaló Z, Gatermann R, Redekop W. Challenges in Research and Health Technology Assessment of Rare Disease Technologies: Report of the ISPOR Rare Disease Special Interest Group. Value Health. 2018 May;21(5):493-500. doi: 10.1016/j.jval.2018.03.004	To develop for the first time a catalogue of primary impediments to rare diseases research and HTA	**Challenges in HTA:**<br>▪ Lack of sufficient and robust clinical data<br>▪ No established standard of care<br>▪ Insufficient knowledge of the natural history of the diseases<br>▪ Lack of validated instruments to assess efficacy and effectiveness end points<br>▪ Application of incremental cost-effectiveness ratio threshold			
Sarri G, Rizzo M, Upadhyaya S, Paly VF, Hernandez L. Navigating the unknown: how to best 'reflect' standard of care in indications without a dedicated treatment pathway in health technology assessment submissions. J Comp Eff Res. 2024 Feb;13(2):e230145. doi: 10.57264/cer-2023-0145	Challenges and HTA considerations for new technologies in indications without established clinical pathway	**Challenges:**<br>▪ To systematically identify the comparator evidence and the need to use patient proxies<br>▪ To collect de-novo evidence on a wide range of potential comparators<br>▪ To resolve uncertainty on potential comparators through evidence generation<br>▪ International "postal code" lottery in evidence requirements and health technology assessment acceptance criteria<br>▪ To identify clinical experts and to achieve clinical agreement to inform the standard of care			

File S2.

Literature review

B. What specific issues, beyond those addressed in the EUnetHTA Core Model®, should be considered in the ethical, legal, and social evaluation of AI-driven technologies?

Research question

The research question was reformulated using the PICO model, which includes the study population (P), the intervention being evaluated (I), the comparator (C), and the outcome of interest (O). Table S4 describes the PICO model underlying this research.

Table S4 – PICO model

**Population**	Not relevant
**Intervention(s)**	AI-driven technologies
**Comparator(s)**	Not relevant
**Outcome (s)**	Ethical issues
	Legal issues
	Social issues

Search

We searched PubMed/MEDLINE for articles indexed, using the defined search string based on the outlined PICO model. We developed a search string comprising three semantic clusters: the first cluster addresses the topics of methodology, the second focuses on AI-driven technologies, and the third pertains to ethical, legal, and social issues. The complete search string is presented in Table S5.

Table S5 – Search string

(((Framework* OR method* OR methodology OR guideline*) AND ("Technology Assessment, Biomedical"[Mesh])) AND ("Algorithms"[Mesh])) AND (ethic* OR social OR legal OR juridical)

Inclusion and exclusion criteria

The documents identified through the search strategy were considered eligible unless they met one or more of the following exclusion criteria:

- duplicate studies;
- studies involving a technology not under investigation;
- studies concerning health conditions not under investigation;

- studies involving a population not under investigation;
- study types not relevant for the analysis;
- insufficient information reported in the study on any of the investigated aspects;
- abstract/full-text not available;
- study not available in English.

The reference list of the included documents was manually checked for additional relevant studies. We did not assess the methodological quality of the included studies.

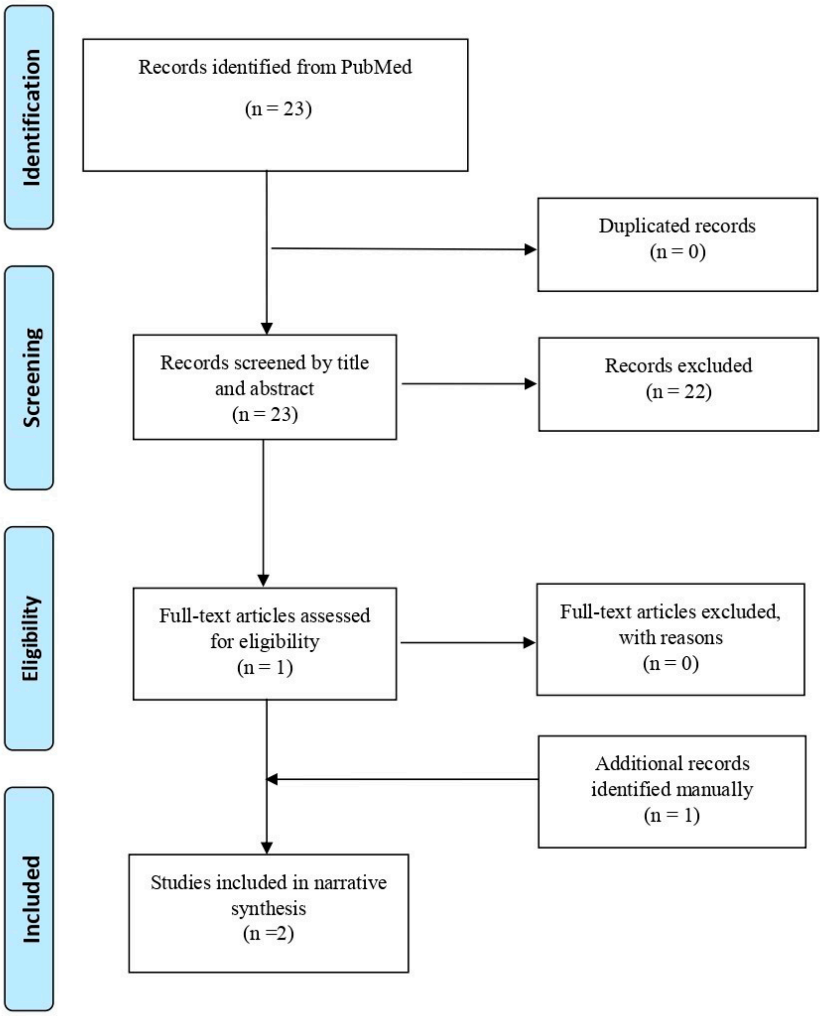

Figure S2 – Study selection

Table S6 – Data extraction

Source	Focus	General considerations	Ethical issues	Legal issues	Social issues
Farah L, Borget I, Martelli N, Vallee A. Suitability of the Current Health Technology Assessment of Innovative Artificial Intelligence-Based Medical Devices: Scoping Literature Review. J Med Internet Res. 2024 May 13;26:e51514. doi: 10.2196/51514	The objective is to critically assess the comprehensive suitability of the current HTA process for AI-based medical devices. This review aims to inform health care professionals, policy makers, and researchers about the challenges and opportunities associated with these technologies.		- The HTA should evaluate whether AI-based medical devices adhere to strict data protection regulations, maintain patient confidentiality, and obtain appropriate informed consent for data use. It is essential to assess whether the devices have mechanisms in place to handle sensitive patient information securely and protect it against unauthorized access or data breaches<br>- the HTA should assess whether the devices have been evaluated for fairness and bias and consider the steps taken to mitigate any identified biases<br>- the HTA should examine the impact of AI-based medical devices on health care disparities and access to care<br>- the HTA should evaluate whether the AI devices provide clear explanations for their outputs and ensure that health care professionals and patients can understand and challenge	- It is necessary to evaluate whether roles and responsibilities of health care professionals complement or replace health care professionals' expertise and whether additional training, supervision, or support is required for their optimal use<br>- it is essential to evaluate whether these devices adhere to relevant data protection laws, such as the General Data Protection Regulation in the European Union<br>-it is crucial to assess the security measures implemented to prevent unauthorized access, data breaches, or tampering<br>- Liability is also a significant legal aspect to be addressed in the context of AI-based medical devices<br>- AI devices need to undergo regulatory approval processes before being introduced into the market	- The HTA should evaluate whether AI-based medical devices adhere to strict data protection regulations, maintain patient confidentiality, and obtain appropriate informed consent for data use. It is essential to assess whether the devices have mechanisms in place to handle sensitive patient information securely and protect it against unauthorized access or data breaches<br>- the HTA should assess whether the devices have been evaluated for fairness and bias and consider the steps taken to mitigate any identified biases<br>- the HTA should examine the impact of AI-based medical devices on health care disparities and access to care<br>- the HTA should evaluate whether the AI devices provide clear explanations for their outputs and ensure that health care professionals and patients can understand and challenge

			the device's recommendations<br>- the HTA should evaluate the extent to which AI-based medical devices can provide interpretable and understandable explanations of their decision-making processes<br>- the HTA should assess whether the device manufacturers provide clear documentation and information to health care professionals and patients about the device's capabilities, limitations, and potential errors		the device's recommendations<br>- the HTA should evaluate the extent to which AI-based medical devices can provide interpretable and understandable explanations of their decision-making processes<br>- the HTA should assess whether the device manufacturers provide clear documentation and information to health care professionals and patients about the device's capabilities, limitations, and potential errors
Farah L, Davaze-Schneider J, Martin T, Nguyen P, Borget I, Martelli N. Are current clinical studies on artificial intelligence-based medical devices comprehensive enough to support a full health technology assessment? A systematic review. Artif Intell Med. 2023 Jun;140:102547. doi: 10.1016/j.artmed.2023.102547	This study aims to investigate whether current studies assessing AI contain the information required for health technology assessment (HTA) by HTA bodies	The current HTA frameworks are not specifically tailored to evaluate AI-based medical devices; however, a core HTA can serve as a foundation for generating specific HTA reports. Our review indicates that the existing HTA frameworks need to be modified to properly assess AI-based medical devices by incorporating factors such as interpretability, explainability, cybersecurity, clinical safety of algorithm updates, interoperability, professional and patient acceptance, as well as ethical and legal considerations. Nevertheless, precise criteria for evaluating AI-			

		based medical devices in the HTA process are still lacking			
Fasterholdt I, Kjølhede T, Naghavi-Behzad M, Schmidt T, Rautalammi QTS, Hildebrandt MG, Gerdes A, Barkler A, Kidholm K, Rac VE, Rasmussen BSB. Model for Assessing the value of Artificial Intelligence in medical imaging (MAS-AI). Int J Technol Assess Health Care. 2022 Oct 3;38(1):e74. doi: 10.1017/S0266462322000551	Model for ASsessing the value of Artificial Intelligence in medical imaging		- **Beneficence and patient integrity:** Address the risk of overdiagnosis, and false-positive and negative results, which may raise a concern or unnecessary invasive procedures. Similarly, consider whether AI predictions indicating low-probability disease risks may cause undue concern for the patient. Further, assess the risk of misdiagnosis and patient harm in the model development phase by carefully curating training datasets and test datasets aligned with recommended standards. In the deployment phase, it is equally important to monitor the AI application's performance<br>- **Privacy:** Consider how to protect patients' right to privacy by using GDPR as a suitable instrument to regulate AI (in tandem with the upcoming EU AI Regulatory Framework), data ownership, consent models, data management, and data security. Furthermore, it is essential to establish guidelines and frameworks for using clinical data for research and development	• Is the Risk Impact Assessment done?<br>• Is the AI model MDR compliant – look after the CE-mark?<br>• Is the Data Protection Impact Assessment (DPIA) done?<br>• Is the mapping of the legal landscape in place?<br>• Is the dataflow in place?<br>• Is the technical description in place?<br>• Are the regulatory approvals in place? (if relevant)<br>• Is there a plan for the ongoing work with legal compliance?<br><br>**Brief description**<br>With relevant legal counseling, map the legal landscape for the entire lifecycle of the AI application<br>• Are the legal requirements (the legal landscape) transformed into functionalities in the AI application?<br>• Is the AI application CE-marked following the MDR regulation?	**Important outcome measures which need to be considered:**<br>• Patient involvement in decision making<br>• Explainability of AI-based projects to the patients<br>• Using appropriate (preferably online) surveys for patients' feedback on their experience<br>• Feasibility to reach patient-friendly imaging reports<br>• Patients' access to imaging data<br>• Using standard patient satisfaction score for satisfaction criteria<br>• Considering patients with special medical conditions<br>• Considering imaging time and time required to receive imaging report<br>• Considering socioeconomic features of the target patient population<br>• Development of patient-friendly software/application for remote access<br>• Fulfill the patients' expectations regarding the use of the latest and greatest technology

			- **Equity (fairness):** Assess if the AI application promotes equity and diversity by facilitating equitable use and access to AI healthcare applications. In the model development and deployment phase, consider the risk of culturally biased health data, which may negatively impact patient groups (e.g., AI skin cancer diagnostic systems trained on white people may misdiagnose other skin colours). Likewise, consider the overall risk of stigmatization or discrimination toward specific patient groups due to encoded bias (concerning, e.g., gender, ethnicity, and religion).<br>- **Autonomy. Trust:** It is essential that AI-enhanced clinical decision-making is trustworthy. To assess the interplay between clinical practice and AI concerning trust, consider the risk of lack of confidence in AI diagnostics by paying attention to transparency, accountability and responsibility issues. But also, consider privacy, e.g., that patient confidentiality is not threatened by AI, which may otherwise cause distrust in the healthcare system and healthcare		**Upcoming challenges:**<br>• When and at which level should patients be informed about the involvement of AI?<br>• Should it always be a free choice to be assessed by AI?<br>• Taking into account the different patient wishes<br>• Changing the patient's view to accept the use of AI instead of doctors<br><br>**Brief description**<br>• Patients' willingness and satisfaction (e.g., effects on subscales for patient satisfaction)<br>• Technical improvement during the imaging process (e.g., shortening scanning time)<br>• Clinical-based patient benefits (e.g., ensuring earlier diagnosis, continuous monitoring)<br>• Overall patient and social benefits

			professionals;<br>**Transparency**.<br>Transparency is essential to validate results and to justify and legitimize decision-making. In the AI model development phase, assess the level of model interpretability, i.e., the degree to which a skilled AI developer understands the model's output at the technical level. In the AI deployment phase, assess AI explainability, i.e., does the AI system design have an explainable interface that conveys understandable information?<br><br>**Brief description**<br>• Is the AI application integrating Ethics by Design?<br>• Beneficence and patient integrity (e.g., risk of over-diagnosis, risk of misdiagnosis/patient harm)<br>• Privacy (e.g., patient confidentiality)<br>• Equity (e.g., equitable use and access to AI applications)<br>• Trust, transparency, accountability, and responsibility (risk of lack of confidence in the AI)<br>• Autonomy (e.g., ensure human oversight and control of AI applications)		

Alami H, Lehoux P, Auclair Y, de Guise M, Gagnon MP, Shaw J, Roy D, Fleet R, Ag Ahmed MA, Fortin JP. Artificial Intelligence and Health Technology Assessment: Anticipating a New Level of Complexity. J Med Internet Res. 2020 Jul 7;22(7):e17707. doi: 10.2196/17707		▪	• When is AI considered as a decision-making support tool? When is it considered as a decision-making tool?<br>• What are the limits of technology and their potential legal implications?<br>• If the AI makes a mistake (eg, black box), who will be held responsible? If the patient is harmed, who will pay for the repairs?<br>• What would be the consequence if the clinician does not comply with the recommendations of an AI and this leads to an error?<br>• AI needs access to data from different sources: consent is becoming more complex, as patients will be asked to authorize the use of diversified<br>• Protection and confidentiality: origin of the data, how consent was obtained, and authorization to use and/or reuse the data<br>• Who owns the data? Who is responsible for it? Who can use (or reuse) it and under what conditions?	When is AI considered as a decision-making support tool? When is it considered as a decision-making tool?<br>• What are the limits of technology and their potential legal implications?<br>• If the AI makes a mistake (eg, black box), who will be held responsible? If the patient is harmed, who will pay for the repairs?<br>• What would be the consequence if the clinician does not comply with the recommendations of an AI and this leads to an error?<br>• AI needs access to data from different sources: consent is becoming more complex, as patients will be asked to authorize the use of diversified<br>• Protection and confidentiality: origin of the data, how consent was obtained, and authorization to use and/or reuse the data<br>• Who owns the data? Who is responsible for it? Who can use (or reuse) it and under what conditions?	

File S3.
Focus group with HTA experts
January 29, 2025. Online

Participants
6 experts in ethical, legal, and social assessment within HTA programs.
Countries of origin: Canada (1), Sweden (1), Italy (1), Norway (1), and the Netherlands (2)
2 moderators:
- Pietro Refolo, Università Cattolica del Sacro Cuore
- Dario Sacchini, Università Cattolica del Sacro Cuore

1 rapporteur:
- Costanza Raimondi, Università Cattolica del Sacro Cuore

Objectives
Building on insights from the literature, experts were consulted to:
- identify gaps in the existing research;
- highlight additional relevant aspects based on their professional experience.

Duration
1 hour

Structure of the session
- Introduction of the participants
- Short explanation of the objectives of MELCAYA WP7
- Short explanation of the final objective of UCSC within WP7
- For each research questions, two questions were asked to the participants: 1) what is missing in the literature? 2) based on your experience, is there any issue that you would add?

NOTE: The content of this document reflects individual opinions and personal experiences. It does not represent consensus views or official recommendations from the MELCAYA project.

Synthesis of key points

Ethical considerations in AI and healthcare
Autonomy and Informed Decision-Making: Ensuring that patients have the broadest possible scope for informed choices, including the option to decline AI-based treatments. However, technological complexity often limits the understanding of both patients and, at times, providers.

Patient and Community Involvement: Engaging affected populations in the design and deployment of AI technologies is crucial to avoid biases and ensure equitable outcomes.

AI's Impact on Clinical Expertise: The increasing reliance on AI for decision-making raises concerns about the potential erosion of clinicians' skills and their ability to recognize AI errors.

Defensive Medicine: Ethical concerns related to unnecessary procedures prompted by risk-averse decision-making, with implications for patient safety and resource allocation.

Bias, accountability, and justice

Types of Bias in AI: Discussions emphasized the need to differentiate between social, structural, human, and statistical biases.

Interconnected Ethical Concepts: The relationship between accountability, ownership, and liability should be clarified within the ethical framework.

Environmental Impact: AI-driven healthcare technologies have significant energy demands, raising concerns about sustainability and their weight in HTA evaluations.

Socioeconomic Factors: The role of educational level in accessing and navigating healthcare systems, particularly for rare diseases, was highlighted as an underexplored area.

Methodological and conceptual issues in HTA

Procedural Framework: The clarity of this concept within the document was questioned, suggesting a need for explicit explanations and structured guidance.

Integration of Facts and Values: The document should ensure that ethical implications are consistently connected to empirical findings.

Question Framing in HTA Tools: Questions should encourage critical thinking rather than requiring clinicians to conduct empirical research to validate ethical concerns.

Terminology and policy considerations

Use of the Term "Orphan Devices":

Some experts noted that while the analogy with orphan drugs is useful, there are significant differences between market dynamics of drugs and devices.

Concerns were raised about whether AI technologies for rare diseases should fall under this classification.

The potential for policy developments, such as an "Orphan Device Act" was mentioned as a consideration for future regulatory discussions.
